# Germinal center‐derived lymphomas: The darkest side of humoral immunity

**DOI:** 10.1111/imr.12755

**Published:** 2019-03-15

**Authors:** Coraline Mlynarczyk, Lorena Fontán, Ari Melnick

**Affiliations:** ^1^ Department of Medicine Division of Hematology & Medical Oncology Weill Cornell Medicine New York City New York

**Keywords:** epigenetic deregulation, germinal center, immune surveillance, lymphomagenesis, precision therapy, signal transduction

## Abstract

One of the unusual features of germinal center (GC) B cells is that they manifest many hallmarks of cancer cells. Accordingly, most B‐cell neoplasms originate from the GC reaction, and characteristically display abundant point mutations, structural genomic lesions, and clonal diversity from the genetic and epigenetic standpoints. The dominant biological theme of GC‐derived lymphomas is mutation of genes involved in epigenetic regulation and immune receptor signaling, which come into play at critical transitional stages of the GC reaction. Hence, mechanistic studies of these mutations reveal fundamental insight into the biology of the normal and malignant GC B cell. The BCL6 transcription factor plays a central role in establishing the GC phenotype in B cells, and most lymphomas are dependent on BCL6 to maintain survival, proliferation, and perhaps immune evasion. Many lymphoma mutations have the commonality of enhancing the oncogenic functions of BCL6, or overcoming some of its tumor suppressive effects. Herein, we discuss how unique features of the GC reaction create vulnerabilities that select for particular lymphoma mutations. We examine the interplay between epigenetic programming, metabolism, signaling, and immune regulatory mechanisms in lymphoma, and discuss how these are leading to novel precision therapy strategies to treat lymphoma patients.

## 1 INTRODUCTION

**Figure 1:**
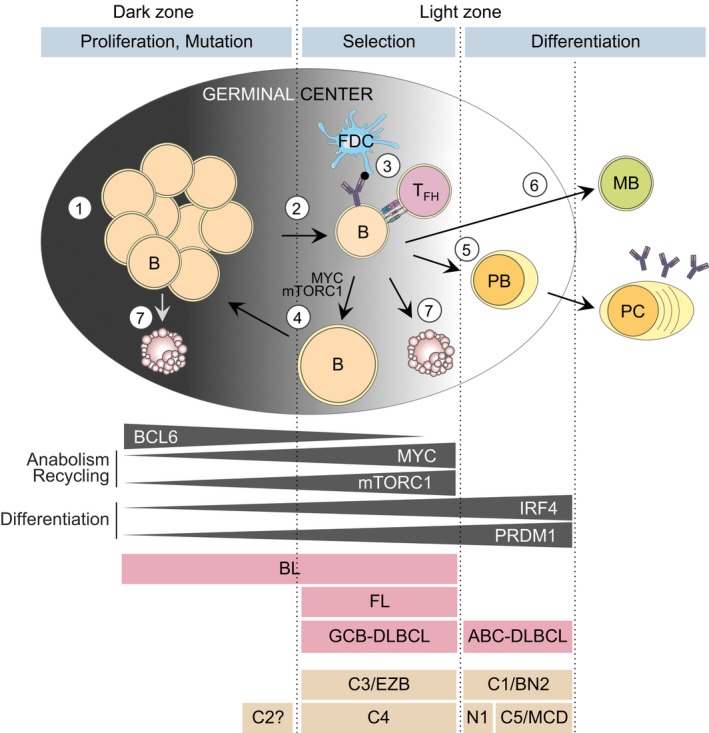
Cell fate decisions in the germinal center (GC) and derived lymphomas. Transient GCs are formed by antigen‐activated mature B cells. (1) In the dark zone, GC B cells proliferate and undergo somatic hypermutation to mutate their immunoglobulin genes. (2) GC B cells transit from the dark zone to the light zone after having divided a determined number of times. (3) In the light zone, cells are selected based on their affinity for the encountered antigen, through their interaction with follicular dendritic cells (FDCs) and follicular T‐helper cells (T_FH_). (4) After positive selection, cells can concomitantly activate MYC and mTORC1 anabolic programs to grow in size and recycle back to the dark zone for further mutation and clonal expansion. (5) Selected cells can also exit the GC and differentiate into plasma cells (PC) via the intermediary plasmablast (PB) stage, or (6) differentiate into memory B (MB) cells. (7) Cells that are not selected in the light zone or that are damaged during somatic hypermutation in the dark zone undergo apoptosis. Mutual exclusion of BCL6 expression or activity with MYC/mTORC1 reflects commitment to proliferation vs anabolism and recycling. With IRF4/PRDM1 it reflects maintenance of GC identity vs GC exit through differentiation into PC. The putative relation of GC‐derived B‐cell lymphomas and DLBCL subtypes with their respective GC cell‐of‐origin, based on transcriptional and genetic profiles and other characteristics, is shown. Note that C1/BN2 cases resemble marginal zone B cells and that the C5/MCD cases are similar to the extranodal forms of DLBCL

Germinal centers (GC) are transient and dynamic structures that form within lymphoid follicles upon antigen challenge.^1^ Antigen‐activated GC B cells undergo rapid proliferation and somatic hypermutation of their immunoglobulin variable genes in order to generate high‐affinity B‐cell receptors (BCR). These densely packed replicative B cells form the GC dark zone. Post‐replicative GC B cells migrate to the more heterogeneous milieu of the GC light zone, where they interact with T follicular helper (T_FH_) and follicular dendritic cells (FDCs). This process leads to the selection of GC B cells that can differentiate into antibody‐secreting plasma cells or memory B cells, to build an immunological memory for future antigen recalls (Figure 1). A small subset of high‐affinity light zone GC B cells recycle back to the dark zone for additional rounds of somatic hypermutation and proliferation, whereas the majority of low‐affinity GC B cells undergo apoptosis.

The GC B cell is at a particularly high risk for undergoing malignant transformation, due to attenuation of certain DNA damage and cell proliferation checkpoints, which is essential for immunoglobulin affinity maturation. Although the GC reaction is tightly regulated, somatic hypermutation can disrupt this delicate equilibrium by generating off‐target mutations that enable B cells to gain selective advantages. Along these lines, a majority of healthy individuals are believed to harbor premalignant clonal populations of mutant B cells, although at the current time there is no way to identify who is at risk for transforming into overt disease.

Germinal center‐derived lymphomas are markedly heterogeneous, as befitting a tissue of origin that naturally undergoes rapid clonal diversification. They are among tumors with the highest burden of somatic mutations, and physically manifest in very diverse manners. Because of this, the classification schemes for these tumors have been in constant evolution, as improving technologies allow deeper insight into their genetic and epigenetic features. This, combined with major advances in understanding GC biology, including at the epigenetic, metabolic, signaling, and immune synapse levels, have revealed how intricate GC regulatory processes can be hijacked in a multitude of ways to facilitate lymphomagenesis. Importantly, these discoveries point toward novel therapeutic paradigms for controlling and even curing lymphomas that were formerly refractory to existing treatments.

## 2 GC‐DERIVED B‐CELL LYMPHOMAS

A majority of non‐Hodgkin lymphomas (NHL), which represent the fifth most common cancer in the United States, arise from B cells transiting the GC reaction. Those that most closely reflect the biology of GC B cells include: (a) diffuse large B‐cell lymphomas (DLBCLs), which are aggressive and rapidly progressing diseases and the most frequent form of NHL. Standard treatment involves combination of anti‐CD20 monoclonal antibody with polychemotherapy. Although often successful, approximately 30%‐40% of DLBCL patients either relapse or are refractory to this treatment and die of their disease. (b) Follicular lymphoma (FL) is an indolent tumor usually diagnosed in older individuals. FL patients have a risk of about 3% per year of transforming into an aggressive and refractory form of DLBCL.^2^, ^3^, ^4^ Treatment for FL is often delayed until there is evidence of progression. While highly sensitive to current therapy, these tumors are almost never cured. (c) Burkitt lymphoma (BL) is a rare form of GC‐derived lymphoma that arises mostly in children and young adults. The African endemic form is linked to EBV infection, whereas the sporadic form often develops at extranodal sites and is not necessarily EBV‐related. BLs are highly aggressive, although intensive treatment is generally successful in eradicating this disease.

Diffuse large B‐cell lymphoma, FL, and BL were identified as originating in the GC because their immunoglobulin variable genes carried somatic hypermutation marks, which are characteristic of GC‐experienced cells.^5^, ^6^ They also classically harbor immunophenotypic, histological, and gene expression features that are consistent with a GC B cell‐of‐origin.^7^, ^8^, ^9^, ^10^ Based on transcriptional profiling, BL can resemble dark zone or light zone GC B cells.^11^ DLBCL is typically divided into two subtypes, the GC B cell‐like (GCB‐) DLBCL, which together with FL, is more transcriptionally reminiscent of light zone GC B cells,^11^ and the activated B cell‐like (ABC‐) DLBCL, which is most similar to plasmablasts (Figure 1).^9^, ^12^

## 3 GC B CELLS INHERENTLY FEATURE HALLMARKS OF MALIGNANT TRANSFORMATION

B cells transiting the GC reaction manifest phenotypic features that mimic many of the canonical biological hallmarks of cancer.^13^ Some of these include:

- *Massive proliferation and clonal expansion*: Licensing of cell proliferation is initiated by transient induction of MYC early in the GC reaction, as well as upon receiving strong T_FH_ cell help in the light zone for dark zone reentry and clonal expansion.^14^, ^15^ In the GC dark zone, proliferation is maintained through the checkpoint suppressing actions of the transcriptional repressors EZH2 and BCL6, and through cyclin D3 activation downstream of GSK3.^16^, ^17^, ^18^, ^19^, ^20^
- *Inactivation of tumor suppressors*: To allow cell cycle progression despite high levels of stress, GC B cells downregulate tumor suppressors such as *TP53* as well as cell cycle checkpoint genes (eg, *CDKN1A*,* CDKN1B*).^18^, ^21^
- *Genome instability*: Somatic hypermutation and class‐switch recombination are mediated by activation‐induced cytidine deaminase (AICDA).^22^ The error‐prone DNA polymerase eta (Polη) additionally introduces DNA point mutations when repairing AICDA‐induced lesions.^23^
- *Resistance to DNA damage*: To facilitate somatic hypermutation, GC B cells downregulate major DNA damage sensor and response proteins including ATR, CHEK1, and TP53.^24^, ^25^ Dampening of DNA damage checkpoints combined with AICDA activity puts cells at high risk of accumulating off‐target genetic alterations.
- *Extended replicative potential*: Upregulation of telomerase expression,^26^, ^27^, ^28^ and protection against AICDA off‐target shortening of telomeres by the uracil‐DNA glycosylase (UNG)^29^ provide GC B cells with improved replicative capacity.
- *Metabolic reprogramming*: In the GC, B cells progress through different environmental conditions with varying levels of oxygen and nutrients. They adapt to these conditions by changing their capacity to use distinct energy sources (eg, glucose, glutamine) and to metabolize them (through oxidative phosphorylation, fermentation, or pentose phosphate pathway [PPP]).^30^, ^31^, ^32^
- *Immune evasion*: In the dark zone, immune synapse components including antigen presentation MHC II genes and *PD‐L1* are downregulated^11^ (and unpublished data) to prevent premature suppression of B cells during clonal expansion. These genes are upregulated in the light zone and required for selection and exit from the GC reaction.^33^, ^34^
- *Terminal differentiation blockade*: GC B cells repress the plasma cell regulators *PRDM1* (BLIMP1) and *IRF4* to maintain a mature but undifferentiated, proliferation‐prone state.^21^, ^35^

Many of the somatic mutations occurring in GC‐derived lymphomas have the primary effect of preventing resolution of these high‐risk GC B‐cell features, thus maintaining GC B cells in a pseudo‐transformed state that eventually leads to full malignant transformation.

## 4 CELL FATE DECISIONS DURING THE GC REACTION CREATE VULNERABILITY TO TRANSFORMATION

### 4.1 GC entry

Upon antigen encounter, naive B cells move to the T‐B border of the follicle to interact with CD4+ T cells. There, the duration of their interaction depends on their specificity and affinity for the encountered antigen.^36^ Resulting co‐stimulatory signals induce B‐cell proliferation at the outer B cell follicle and then migration to the center of the follicle to form a nascent GC.^37^ Along these lines, genetic lesions that occur prior to the GC reaction, such as those affecting *TET2* (arising in hematopoietic stem cells) or *BCL2* (in pre‐B cells), may confer preferential initial expansion and survival of mutant cells, resulting in an expanded population of GC B cells at risk for acquiring a “second hit.”^38^, ^39^, ^40^

### 4.2 Dark zone to light zone transition

Germinal center B cells move to the light zone after undergoing a defined number of cell divisions ranging from 1 to 6, depending on several factors including BCR affinity for antigen.^41^, ^42^ Aberrant retention of B cells in the dark zone proliferative stage of development would be expected to foster malignant transformation and an aggressive phenotype. This situation is best represented by BL, which can manifest a gene expression profile similar to dark zone GC B cells.^11^ It is likely that the characteristic *MYC* translocation occurring in these tumors enables sustained proliferation due at least in part to enhanced metabolic sufficiency.

### 4.3 Selection by T_FH_ cells and FDCs

Light zone GC B cells interact with antigen‐coated FDCs through their BCR and seek help from T_FH_ cells via CD40 and MHC II as well as other co‐receptors, which collectively form the “immune synapse.” This selection process is required to maintain survival of high‐affinity B cells and direct them to recycle to the dark zone, or terminally differentiate into plasma or memory B cells (Figure** 1). BL, FL, and DLBCL largely represent GC or post‐GC B cells, in which these immune signals were either disrupted or amplified due to the actions of specific somatic mutations. Genetic lesions that disrupt these signals (eg, loss‐of‐function of MHC II, MHC I, and epigenetic modifiers like *KMT2D* and *CREBBP*) can promote T cell‐independent survival and failure to exit the GC reaction. Conversely, somatic mutations that induce constitutive activation of the BCR and other immune synapse genes (eg, those affecting *CD79B*,* CARD11*, or *MYD88*) can maintain survival and proliferation signals normally linked to early plasma cell development.

### 4.4 Cyclic reentry to the dark zone

About 10%‐30% of B cells possess sufficient antigen affinity to evade apoptosis but yet do not differentiate into plasma cells or memory B cells and instead reenter the dark zone for further rounds of somatic hypermutation and proliferation (Figure** 1).^14^, ^15^, ^42^, ^43^, ^44^, ^45^ Cooperation of CD40 and BCR signaling activated in an affinity‐dependent manner leads to the induction of MYC^46^ and mTORC1.^47^ Transient MYC‐ and mTORC1‐activation enables light zone cells to become anabolic and generate the pools of metabolic precursors that will be required for proliferation in the dark zone. Normally, this process is strictly compartmentalized so that GC light zone cells do not proliferate while they undergo MYC/mTORC1‐dependent anabolic charging (Figure** 1). In contrast, in the GC dark zone MYC is silenced and mTORC1 activity reduced, which may limit the ability of rapidly dividing cells to undergo continuous replication. In part, this separation of anabolism and proliferation is due to the actions of the transcriptional repressor BCL6, which simultaneously silences MYC and multiple cell cycle checkpoint genes.^48^ Any loss of this MYC‐BCL6 mutual exclusivity is inherently dangerous and could unleash unlimited proliferation, as occurs in DLBCL and BL.

### 4.5 GC exit to plasma cell differentiation

Commitment to the plasma cell fate is associated with highly stable B‐T_FH_ contacts and requires robust CD40 signaling and transcriptional reprogramming.^49^ Disruption of this process can favor the accumulation of proliferating plasmablastic cells. This effect appears to underlie the tumorigenic effect of mutations that affect the transcription factor *PRDM1* (BLIMP1), which is the master regulator of plasma cell differentiation.^50^ Translocations that induce constitutive expression of BCL6 may also lead to aberrant repression of *PRDM1*.^35^, ^51^ *PRDM1* loss occurs almost exclusively in patients with ABC‐DLBCLs, many of which manifest a plasmablastic transcriptional profile (Figure** 1).

### 4.6 GC exit to memory B‐cell differentiation

Memory B cells begin to generate early in the GC reaction and manifest lower immunoglobulin affinity as compared to plasma cells that egress later.^52^ Upon antigen recall, memory B cells will either differentiate into antibody‐producing cells or reseed new GCs to undergo further immunoglobulin affinity mutation. Memory B cell reentry into de novo GCs may be critical for the development of B‐cell lymphomas, as this allows for repeated rounds of mutagenesis to occur in GC B cells already bearing founder mutations. Along these lines, memory B cells carrying the FL hallmark *BCL2/IGH* t(14;18) translocation, which constitutively activates the anti‐apoptotic *BCL2* gene, preferentially reenter GCs after repetitive immunological challenge as compared to normal memory B cells and mediate progression to FL‐like stages.^40^ Multihit lymphomagenesis may therefore occur over time through recurring GC transits.

### 4.7 Cell death

Up to half of all GC B cells are lost through apoptosis every six hours during the GC reaction.^53^ Programmed cell death can occur by default for cells that are not positively selected.^53^ For example, GC B cells can be poised to undergo apoptosis due to silencing of *BCL2* by the BCL6 transcriptional repressor. In lymphoma, *BCL2* translocations bypass this effect by driving *BCL2* expression through alternative immunoglobulin enhancers and promoters. With acquired resistance to apoptosis, *BCL2*‐translocated tumors manifest as gradually accumulating B cells within follicular structures. In addition to death by “neglect” in the light zone, GC B cells undergo apoptosis in the dark zone if they are damaged during somatic hypermutation.^53^, ^54^ DNA damage can downregulate BCL6 and restore DNA damage checkpoint activity.^55^ However, somatic mutations that disrupt reexpression of BCL6‐repressed targets (such as mutations affecting *CREBBP* or *EP300*) or that result in constitutive BCL6 expression (eg, *3q26* translocations) may enable these damaged cells to escape apoptosis.

## 5 THE GENETIC BASIS OF GC‐DERIVED B‐CELL LYMPHOMAS

Germinal center‐derived B‐cell lymphomas have been defined (DLBCL, FL, and BL) based on histopathology and gene expression profiles.^7^, ^8^, ^9^, ^10^ DLBCL has been further classified into the GCB‐ and ABC‐DLBCL subtypes based on gene expression.^9^ An additional primary mediastinal subtype of DLBCL tends to occur in younger individuals with a bias toward females, and features strong NF‐κB signaling signatures.^56^ However, in the genome‐sequencing era it has become evident that these diseases are far more intricate. Indeed, GC‐derived lymphomas are among the most genetically complex and heterogeneous of all tumor types. It is estimated that there are ~150 highly recurrently mutated genes (defined as occurring in >5% of patients) in DLBCL.^57^ There is also a heavy burden of copy number and structural variations in these tumors.^58^ All of this is reasonable to expect, given the inherent mutability of their cell‐of‐origin. Strikingly, more than 50% of genes mutated in DLBCL are transcription factors or chromatin modifiers.^57^, ^59^ There are frequent mutations of immune signaling pathway genes including antigen presentation, BCR signaling, NF‐κB signaling, PI3K, Toll‐like receptor (TLR), and NOTCH signaling. Table** 1 provides an integrated and curated list of genes that are recurrently mutated in BL, FL, and DLBCL subtypes; Table** 2 gives an overview of lymphoma mutated genes, for which mechanistic studies have yielded insight into their role in the GC reaction and lymphomagenesis.^60^, ^61^, ^62^, ^63^, ^64^, ^65^, ^66^, ^67^, ^68^, ^69^, ^70^, ^71^, ^72^, ^73^, ^74^, ^75^, ^76^, ^77^, ^78^, ^79^, ^80^

**Table 1:**
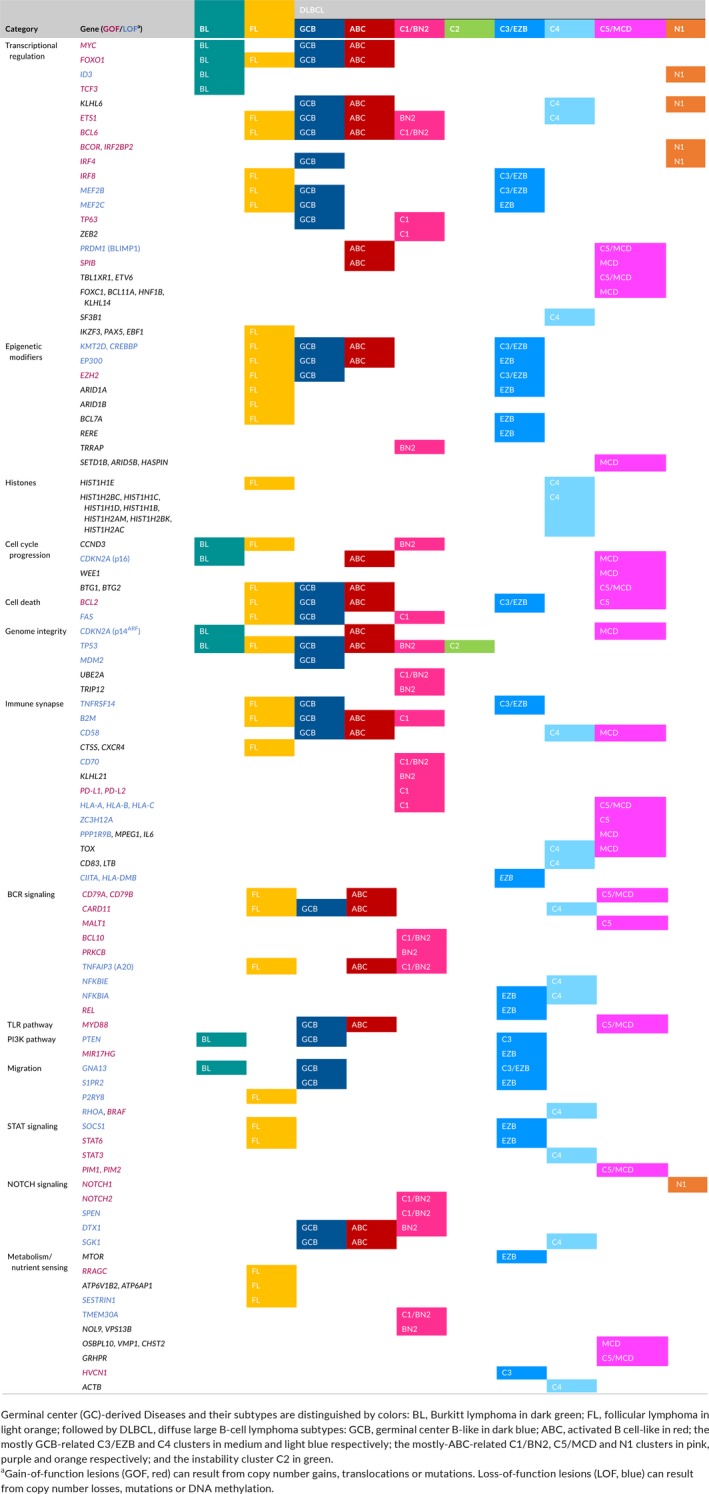
Summary of genes affected in GC‐derived B‐cell lymphomas across disease subtypes and grouped in biological categories

**Table 2:**
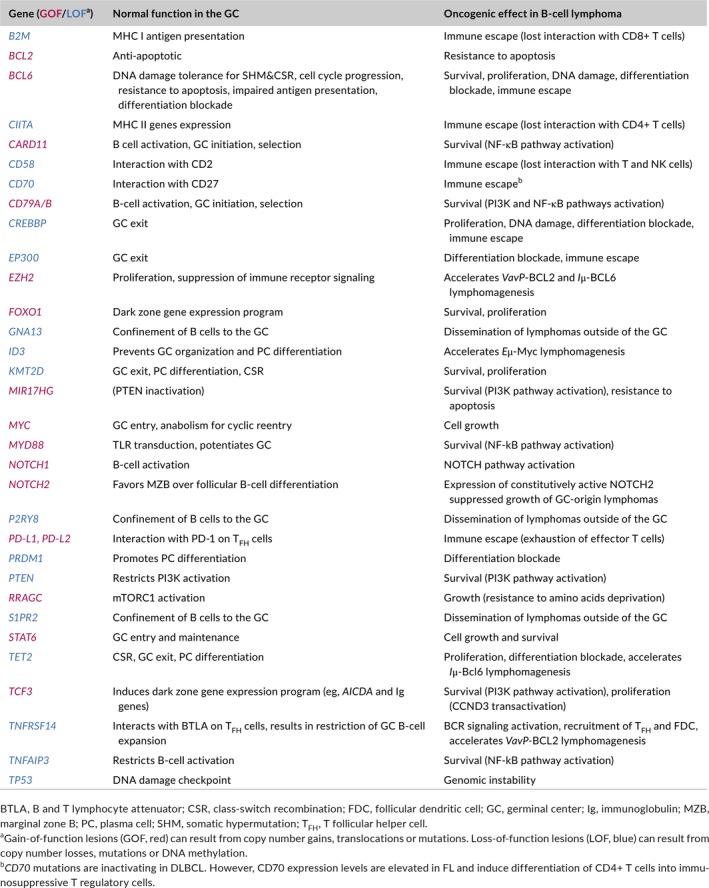
Oncogenic effects of genetic lesions found in GC‐derived B‐cell lymphomas and corresponding biological functions in the GC

Recent efforts have attempted to improve the molecular classification of DLBCL through integrative analysis of their genomic profiles.^58^, ^81^ These studies included recurring mutations, copy number variations, and chromosomal rearrangements (Table** 3). Chapuy et al identified five clusters, C1‐C5, of which two are GCB‐ and two are ABC‐related subtypes, each with differences in clinical outcome and possible therapeutic vulnerabilities. The fifth cluster is mostly characterized by genomic instability and *TP53* mutations.^58^ Schmitz et al defined four different subsets named BN2, MCD, N1 (these three are mostly ABC), and EZB (mostly GCB) (Table** 3). The four subtypes were also associated with distinct therapeutic responses, gene expression signatures, and targetable pathways.^81^ In the C1‐C5 classification, the unclassified cases (C0) represent less than 4% of the n = 304 DLBCL cases, while in the EZB/BN2/MCD/N1 classification, more than 50% of DLBCL cases are predicted to remain unclassified. This discrepancy might be due, at least in part, to the fact that the latter was generated based on a cohort enriched for ABC and COO‐unclassified cases, and that copy number variations in the C1‐C5 classification seemed to be more extensively analyzed.

**Table 3:**
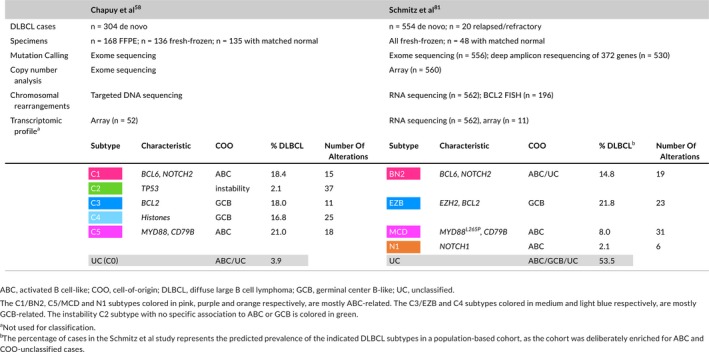
Comparison of the genomic profile‐based DLBCL molecular classifications

More specifically, the C1 and BN2 subtypes (~18% and 15% of all DLBCLs, mostly ABC) are characterized by *B* *CL6* translocations and *N* *OTCH* *2* activating mutations. Out of 15 genetic drivers in C1 DLBCLs, 7 also define BN2, corresponding to a 47% “overlap” in affected genes. Given the role of NOTCH2 as a driver of marginal zone B‐cell phenotype and the presence of additional mutations linked to non‐canonical NF‐κB signaling, these lymphomas are proposed to be of potential marginal zone B‐cell origin, and represent a novel group of ABC‐DLBCL with favorable clinical outcome.

The C5 and MCD (~21% and 8% of all DLBCLs) are also part of the ABC spectrum and are strongly enriched for *M* *YD88* L265P mutations and *CD* *79B* mutations and amplifications. Seven out of the 18 C5‐defining genetic alterations are also found in MCD, corresponding to about 40% “overlapping” genes. These cases manifest increased extranodal involvement, are genetically similar to extreme extranodal forms of DLBCL such as primary central nervous system lymphoma (PCNSL)^81^ and contribute to the inferior outcome that was previously noted in ABC‐DLBCL cases.

The N1 subtype is a less represented ABC subtype (2.1%) mostly defined by *N* *OTCH* *1* mutations and associated with poor prognosis.

The C3 and EZB clusters represent a majority of GCB‐DLBCLs and include 18% and 21.8% of all DLBCLs, respectively. These cases are characterized by *B* *CL2* translocations, mutations in the chromatin modifiers *EZ* *H2*,* CREBBP*, and *KMT2D*, loss of *TNFRSF14* expression, and PTEN inactivation. These genetic lesions also occur in FL and may be consistent with their shared light zone origin.

The C4 cluster (16.8% of DLBCLs) also includes GCB cases and manifests high frequency of histone mutations. Genetic lesions of histone genes do not feature prominently in the EZB/BN2/MCD/N1 classification perhaps due to distinct approaches in somatic mutation calling and in distinction of clonal vs subclonal mutations. Both C4 and EZB subtypes feature mutations targeting the NF‐κB and JAK/STAT signaling pathways, and C4 cases show better clinical outcome than C3 cases.

The genome instability cluster C2 (2.1% of DLBCLs) is defined by the presence of *TP53* mutations, copy number variations, and an increase in ploidy. It contains both ABC and GCB cases.

From the genetic perspective, FL cases, like the C3/EZB DLBCLs harbor *BCL2* translocations to the *IGH* locus that are believed to occur prior to GC formation during VDJ recombination. The majority of FL cases carry mutations in at least one of the following histone modifiers: *CREBBP*,* KMT2D*,* EZH2*, or *EP300*, and they typically fail to respond to GC exit signals.^82^

Burkitt lymphoma is characterized by *MYC* translocation to the immunoglobulin loci resulting in MYC overexpression. BL cases also present activating mutations of *TCF3* (11%) and loss of its negative regulator *ID3* (58%) that activate “tonic BCR signaling.”^83^ Moreover, *PTEN* is mutated in 7% of BL patients and PTEN*‐*targeting *MIR17GH* is overexpressed.^83^

The following sections illustrate some of the biological mechanisms and therapeutic vulnerabilities induced by lymphoma‐associated somatic mutations.

## 6 BCL6 AND CONTROL OF THE GC PHENOTYPE

Any discussion of GC mutations and lymphomagenesis requires special consideration of the BCL6 transcriptional repressor, a critical master regulator of the GC phenotype. Expression of BCL6 is required for GC formation, and its actions are dependent on its direct repression of >1000 target genes (reviewed in^84^). Although first cloned as being frequently translocated in B‐cell lymphomas and linked to the GC reaction, BCL6 is in fact widely expressed in many cell types. Evolutionarily, the first recognizable *BCL6* gene appeared over 500 million years ago in early vertebrates.^85^ Functional studies suggest that the ancestral role of BCL6 was to enable vertebrate cells to adapt to stress conditions, downstream of heat shock factor 1 (HSF1).^85^ Presumably, the existence of BCL6 as an inducible stress tolerance protein facilitated evolution of the humoral immune response, where B cells must be able to tolerate the major stresses associated with massive proliferation and mutagenesis.^85^ Lymphoma cells inherit dependency on BCL6 from their GC cell‐of‐origin and are almost universally dependent on BCL6 for their survival and proliferation.^84^, ^86^

To drive the GC phenotype, BCL6 binds and represses genes linked to DNA damage response (eg, *ATR*,* TP53*, etc.) cell cycle checkpoint control (eg, *CDKN1A*,* CDKN1B*, etc.) and genes involved in GC exit and plasma cell differentiation (eg, *IRF4* and *PRDM1*).^84^ BCL6 also represses many of the genes encoding immune receptors and antigen presentation molecules, or involved in signaling pathways associated with recognition by T_FH_ and FDC and selection in the light zone (eg, MHC class II, *CIITA*, BCR pathway genes, etc.).^87^, ^88^ This massive repression allows B cells undergoing proliferation and somatic hypermutation in the GC dark zone to transiently “fly under the radar” of immune regulatory networks until encountering the light zone microenvironment.

BCL6 functions can be either transiently suspended or permanently suppressed in the light zone, which likely plays a key role in determining the fate of individual GC B cells. For example, CD40 signaling triggers ERK‐mediated phosphorylation and shuttling of the BCL6 partner protein SMRT from the nucleus to the cytoplasm.^24^, ^89^ This enables rapid, but transient reactivation of BCL6 target genes that are repressed through BCL6‐SMRT complexes, including antigen presentation (eg, MHC class II loci) and DNA damage (eg, *ATR*) genes.^24^, ^88^, ^89^ This effect likely facilitates T_FH_‐directed natural selection of high‐affinity light zone GC B cells, and DNA damage checkpoint‐mediated elimination of B cells heavily damaged during the GC reaction. Cells selected to recycle to the dark zone would then reassemble BCL6‐SMRT complexes through as of yet unknown mechanisms. Presumably, other BCL6 functions that are not dependent on SMRT are still operational in these light zone cells and help to maintain their GC identity. In contrast, definitive silencing of *BCL6* is mediated by GC exit regulators such as IRF4 and later PRMD1, as well as through BCL6 protein degradation by FBXO11, to enable full resolution of the GC phenotype and terminal differentiation.^90^, ^91^ These mechanistic considerations help to explain the various ways in which BCL6 functions are reinforced and maintained by lymphoma somatic mutations.

Pharmacologic targeting or genetic downregulation of BCL6 results in the death of DLBCL, BL, and FL cell lines or primary human patient specimens within 48‐72 hours.^86^, ^92^, ^93^, ^94^ It was noted previously that GC B cells manifest many of the biological hallmarks of tumor cells, many of which are driven by BCL6. Along these lines, several DLBCL and FL somatic mutations function at least in part by either maintaining or enhancing BCL6 functionality. For example, somatic loss‐of‐function mutation of the histone acetyltransferase CREBBP prevents reactivation of BCL6‐SMRT repressed genes that would normally occur upon T_FH_ engagement in the light zone.^88^, ^95^ CREBBP may also suppress BCL6 functions by directly acetylating its second repression domain, an effect that is impaired by its mutation.^96^ Gain‐of‐function mutations of the polycomb histone methyltransferase gene *EZH2* reinforce silencing of BCL6 target promoters through accumulation of the H3K27me3 (trimethylation of histone 3 lysine 27) mark.^21^ Mutations affecting the transcription factors MEF2B and PRDM1 disrupt their ability to repress BCL6 expression and loss‐of‐function mutations of FBXO11 stabilize BCL6 protein.^91^, ^97^, ^98^ Notably, *BCL6* translocations occur preferentially in the subclass of ABC‐DLBCL (C1 or BN2) that may derive from BCL6‐negative marginal zone B cells instead of GC B cells.^58^, ^81^ Translocation of *BCL6* may thus be qualitatively different from the mutations described above in causing ectopic expression of BCL6 in a cell type that normally does not express this proto‐oncogene in order to endow them with oncogenic GC features.

Finally, certain canonical lymphoma genetic lesions such as *BCL2* and *MYC* translocations may be explained as a way to bypass their transcriptional repression by BCL6.^48^ This leads to a situation where BCL6 pro‐tumor functions are preserved, while its antitumor functions are abrogated. The importance of the tumor suppressor activity of BCL6 is underlined by the fact that lymphomas with double translocation of *MYC* and *BCL2* are among those with the worst prognosis.^99^ From the therapeutic standpoint, targeting BCL6 is lethal to lymphoma cells, but can also result in induction of target genes like BCL2 resulting in a process described as “oncogene switching.” Along these lines combination of BCL6 and BCL2 inhibitors is highly synergistic in killing lymphoma cells.^100^

## 7 CORE EPIGENETIC MECHANISMS DRIVING GC‐DERIVED LYMPHOMAGENESIS

**Figure 2:**
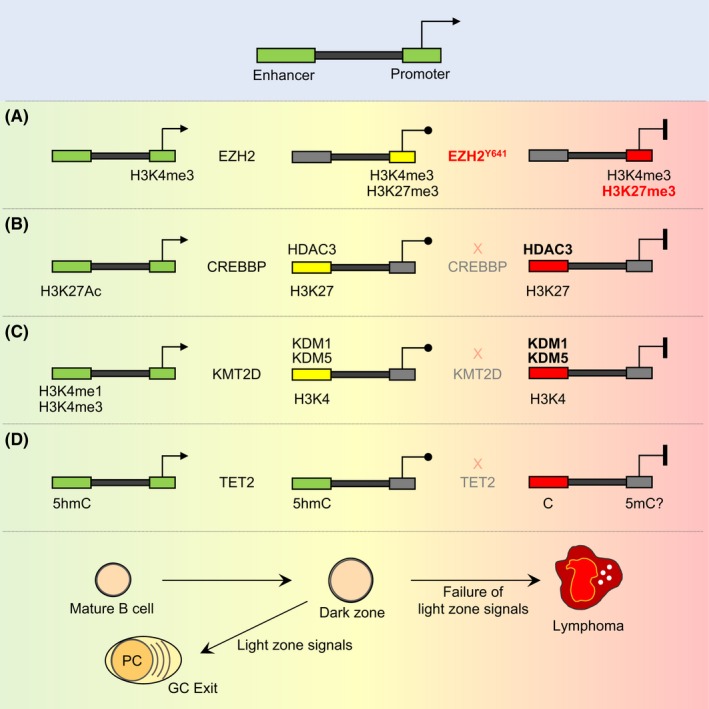
Proposed epigenetic driver mechanisms in GC B‐cell lymphomas. In the GC reaction, there is transient suppression of enhancers and promoters of genes that regulate immune signaling pathways, antigen presentation, and checkpoints, which revert back to the active state when GC B cells are signaled to exit the GC reaction. Lymphomas arise from failure of GC exit signals to restore expression of these genes through several proposed epigenetic mechanisms: (A) EZH2 is induced in GC B cells and converts H3K4me3 active promoters (green) to H3K4me3/H3K27me3 bivalent promoters (yellow) for transient repression of target genes, which is reversed upon GC exit. *EZH2* mutations in lymphoma cause accumulation and permanent silencing (red) of bivalent promoters. (B) CREBBP maintains active enhancers (green) marked by H3K27Ac in mature B cells. In GC B cells, these enhancers are transiently toggled off (yellow) by HDAC3 through H3K27 deacetylation and then restored upon GC exit signaling. In lymphomas with *CREBBP* loss‐of‐function mutation, HDAC3 is now unopposed to maintain aberrant silencing (red) of these enhancers. (C) KMT2D maintains enhancer activity (green) in mature B cells through H3K4me1 and possibly H3K4me3. In the GC, these enhancers become demethylated, possibly through the actions of KDM1 or KDM5 histone demethylases and these enhancers are reactivated in B cells exiting the GC reaction. *KMT2D* loss‐of‐function mutations result in persistent demethylation of enhancers and failure of the respective genes to respond to signals. (D) B‐cell enhancers are decorated by the 5hmC activating mark (green) in a TET2‐dependent manner, which is retained (green) in the GC reaction (unlike the histone marks from B or C). *TET2*‐loss‐of‐function mutation results in failure to maintain enhancer 5hmC and loss of enhancer activating mark H3K27Ac with corresponding repression of the respective genes (red)

During the humoral immune response, GC B cells undergo dramatic and rapid‐sequence phenotypic changes.^101^ Transitioning to the GC phenotype involves deep remodeling of the 3D architecture of the genome and extensive redistribution of epigenetic marks.^87^, ^101^ The GC B‐cell epigenome displays loss of chromatin activating marks and gain of repressive marks at promoters and enhancers for genes involved in cellular checkpoints, BCR signaling, interferon response, antigen presentation, and other mature B‐cell functions.^87^, ^101^ These genes are not “silenced,” but instead are simply held in a poised configuration, where RNA Pol II is present and loaded at promoters but is not actively transcribing nascent mRNAs.^87^, ^101^ This pattern is directed by BCL6 in dark zone GC B cells and lymphoma cells, and is reversible by signals from T_FH_ and FDCs in the light zone. BCL6 mediates this transient repression effect by recruiting (a) SMRT/NCOR‐HDAC3 complexes, (b) BCOR‐RING1B complexes in cooperation with EZH2, and (c) the LSD1 histone demethylase through the BCL6 repression domain 2 (RD2).^21^, ^87^, ^102^ MTA3 and CTBP may also be relevant BCL6 corepressors but this has not been validated outside of the cell line context.^84^ Activation of BCL6 target genes in the light zone is mediated by the histone acetyltransferases CREBBP and EP300, and the histone methyltransferase KMT2D.^88^, ^95^, ^103^, ^104^ Along these lines, FL and GCB‐DLBCL manifest almost universal somatic mutation of chromatin‐modifier genes (eg, *KMT2D*,* CREBBP*,* EZH2*,* TET2*, and *EP300*).^105^, ^106^, ^107^ Taken together, these data indicate that one of the most critical vulnerable transitional states for pathogenesis of GC‐derived lymphomas involves epigenetic remodeling downstream of signals received from T_FH_ and FDCs in the light zone (Figure 2).

Approximately 30%‐40% of patients with DLBCL or FL manifest somatic mutations of *CREBBP*.^88^, ^96^, ^106^ These occur early during pathogenesis and are more frequent in GCB‐DLBCL.^108^ Inactivating missense mutations of the histone acetyltransferase (HAT) domain account for 50%‐75% of cases, whereas most remaining alleles cause truncation or loss of expression.^88^, ^96^, ^106^, ^109^ CREBBP‐mutant FLs manifest strong silencing of antigen presentation genes, lower infiltration of T cells, and reduced ex vivo activation of autologous T cells.^109^ In mice, *Crebbp* loss‐of‐function accelerates lymphomagenesis.^88^, ^95^, ^110^ Crebbp‐deficient murine tumors and human DLBCL cell lines show focal loss of H3K27 acetylation at enhancers and concordant downregulation of antigen presentation and BCR signaling genes.^88^, ^95^ Notably, these are the same genes silenced by BCL6‐HDAC3 complexes in GC B cells, suggesting that HDAC3 is the opposing enzyme to CREBBP and drives the malignant phenotype of CREBBP‐mutant lymphoma cells (Figure** 2).^88^ In line with this, CREBBP‐mutant DLBCL lines are more sensitive to HDAC3 loss than their wildtype counterparts, in vitro and in vivo. EP300 loss‐of‐function also yields a lymphoma tumor suppressor phenotype in mice, and its functions appear to partially overlap with CREBBP in GC B cells.^87^, ^88^

*KMT2D* (*MLL2*) is mutated in 30%‐80% of patients with DLBCL and FL.^96^, ^103^, ^106^ KMT2D is a catalytic component of the COMPASS complex, which induces transcriptional activation through H3K4me1/2 at gene enhancers. Most *KMT2D* lesions are nonsense mutations that truncate the protein upstream of its enzymatic SET domain, thus causing loss‐of‐function.^96^, ^103^, ^106^ *Kmt2d* deficiency results in differentiation blockade, defective class‐switch recombination, aberrant and long‐term persistence of GC B cells, and lymphomagenesis in mice.^103^, ^104^ *KMT2D* mutation or deficiency causes a focal loss of H3K4me1 activating chromatin mark predominantly at enhancers (Figure** 2). This leads to repression or inability to activate genes involved in CD40, BCR, TLR, and other immune pathways.^103^ Importantly, *KMT2D* mutation renders DLBCL cells resistant to CD40 signaling due to suppression of CD40‐responsive enhancers.^103^ Since *KMT2D* mutations result in reduction (but not total ablation) of H3K4 methyltransferase activity in B cells, it is reasonable to hypothesize that loss of enhancer activation due to *KMT2D* mutation is maintained and reinforced by histone demethylases, analogous to the case of CREBBP and HDAC3 on histone acetylation. There are two families of H3K4 demethylases: the KDM1 (LSD1/2) and KDM5 (A/B/C/D) proteins. Although LSD1 is highly upregulated and required for the GC reaction and repression of KMT2D target genes, its methyltransferase function is not required for this effect.^102^ Of the four *KDM5* genes, *KDM5C* and to a lesser extent *KDM5A* are highly expressed in GC B cells, FL, and DLBCL. Their loss‐of‐function was reported to rescue repression of KMT2D target genes in KMT2D‐mutant lymphoma cells and selectively suppress the growth of these cells in vitro and in vivo.^111^

EZH2 is an H3K27 methyltransferase and component of the PRC2 polycomb complex that is upregulated in the GC.^16^, ^112^, ^113^ Conditional deletion of EZH2 in GC B cells results in failure to form GCs.^16^, ^113^ In GC B cells, EZH2 converts gene promoters from an active state marked by H3K4me3 to a bivalent H3K4me3/H3K27me3 “poised” state (Figure** 2).^16^ Repression of these genes requires the presence of BCL6, which together with the H3K27me3 mark recruits the BCOR complex through combinatorial tethering.^21^ Genes regulated in this manner include cell cycle checkpoint genes such as *CDKN1A*, antigen presentation genes, and GC exit genes such as *IRF4*.^16^, ^17^, ^21^, ^114^ It is not yet known how the EZH2 H3K27 methylation program is erased in the light zone, but it is reasonable to postulate that this is essential for GC exit. EZH2 is affected by somatic gain‐of‐function mutations in 30% of GCB‐DLBCL and FL patients.^115^, ^116^ These mutations are always heterozygous and most commonly affect the Y641 residue within the catalytic pocket of the EZH2 enzymatic SET domain. Y641 mutant EZH2 yields more efficient H3K27 trimethylation but loss of H3K27 monomethylation activity. This explains why the mutation is always heterozygous, since the mutant enzyme needs the wildtype protein to monomethylate H3K27.^21^, ^117^ Conditional expression of Y641 mutant EZH2 in GC B cells leads to GC hyperplasia and lymphomagenesis.^16^ EZH2‐specific inhibitors or shRNA cause proliferation arrest and a partial plasma cell phenotype in DLBCL cells, and eventually some degree of apoptosis.^16^ These effects occur more rapidly in cell lines harboring *EZH2* mutations, and justified the clinical translation of EZH2 inhibitors for patients with FL and DLBCL.^16^

Unique among recurrent mutations in DLBCL, *TET2* lesions occur in hematopoietic stem cells and hence are by definition “founder” mutations in DLBCL.^38^ These are generally missense or truncating mutations that result in protein loss.^39^, ^57^ TET2 is an alpha‐ketoglurate (aKG)‐dependent dioxygenase that converts 5'methylcytosine (5mC) into 5'hydroxymethylcytosine (5hmC).^118^ Gene promoter 5mC is linked to transcriptional repression, whereas 5hmC is a transcriptional activation mark at gene enhancers (Figure** 2).^119^ Loss of Tet2 results in GC hyperplasia in mice and failure to undergo class‐switch recombination and plasma cell differentiation, ultimately leading to the development of B‐cell lymphomas.^39^ This phenotype is linked to loss of gene enhancer activating 5hmC and H3K27 acetylation marks in GC B cells, at a similar set of genes that are controlled by CREBBP. *TET2* and *CREBBP* mutations are mutually exclusive in DLBCL, suggesting that they control the same pathways.^39^ Loss of TET2 renders DLBCL cells dependent on HDAC3, suggesting a potential therapeutic approach for TET2‐mutant DLBCL patients.^39^

## 8 DYSREGULATION OF GC METABOLISM

Many B‐cell lymphomas originating in the GC present an exceptionally high proliferation index.^120^ This implies massive metabolic requirements in order to generate sufficient energy and support anabolism for repeated growth and division cycles. Accordingly, lymphoma cells utilize and become dependent on metabolic adaptation mechanisms that are normally only used transiently or not at all by GC B cells. Metabolic perturbations in lymphoma include the deregulation of metabolic checkpoints (eg, mTORC1), the adjustment to low levels of nutrients and oxygen and the rewiring of metabolic pathways to best use available sources.

### 8.1 Aberrant mTORC1 activation

**Figure 3:**
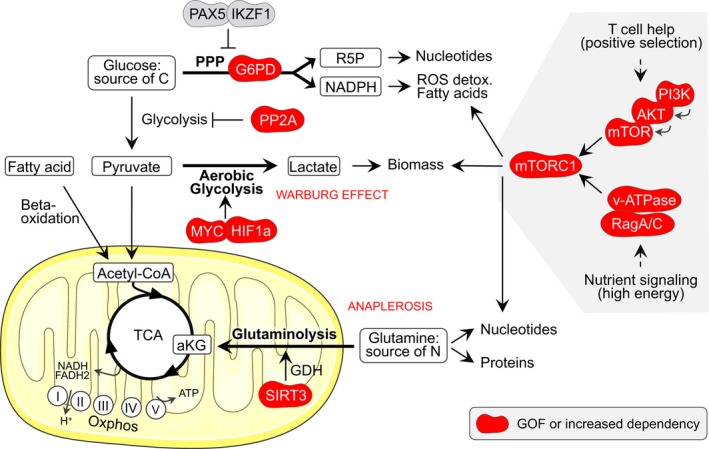
Metabolic dysregulation in GC‐derived B‐cell lymphoma. In the mitochondrion, the tricarboxylic acid (TCA) cycle produces reduced NADH and FADH2 that are used by complexes I to V of the electron transport chain (ETC) to generate ATP through oxidative phosphorylation (oxphos). The TCA can be fueled by fatty acid‐ or pyruvate‐derived acetyl‐CoA. Alternatively, glutamine can be used to generate alpha‐ketoglutarate (aKG). DLBCLs have developed dependency on SIRT3 to replenish the TCA cycle (also known as anaplerosis). SIRT3 stimulates glutaminolysis by activating the glutamine dehydrogenase (GDH). Glucose can be converted into pyruvate through glycolysis or used through the pentose phosphate pathway (PPP) to generate ribose 5‐phosphate (R5P) and NADPH. Some GC‐derived B‐cell lymphomas depend on PP2A and G6PD, a key PPP enzyme, to switch glucose carbon usage from glycolysis to the PPP. This provides antioxidant protection and supports ribonucleotide biosynthesis in proliferating cells. Pyruvate can also be converted into lactate as part of the “aerobic glycolysis” that tumor cells use to “bypass” the TCA cycle, to generate some ATP and to create biomass. This is known as the Warburg effect and can be induced by MYC and HIF1‐alpha stabilization in DLBCL and FL. Finally, mTORC1 activation in the GC happens downstream of T cell‐positive selection signals via the PI3K/Akt/mTOR pathway or downstream of nutrient signaling via activation of RagA/C and the v‐ATPase. These components either carry gain‐of‐function (GOF) mutations, are hyper‐activated, or are expressed at high levels in GC‐derived B‐cell lymphomas, resulting in mTORC1 constitutive activation. mTORC1 is the master regulator of anabolism and while constitutive activation is detrimental to GC B cells, it appears to favor lymphoma growth. G6PD, glucose‐6‐phosphate dehydrogenase; ROS, reactive oxygen species; v‐ATPase, vacuolar ATPase proton pump

**Figure 4:**
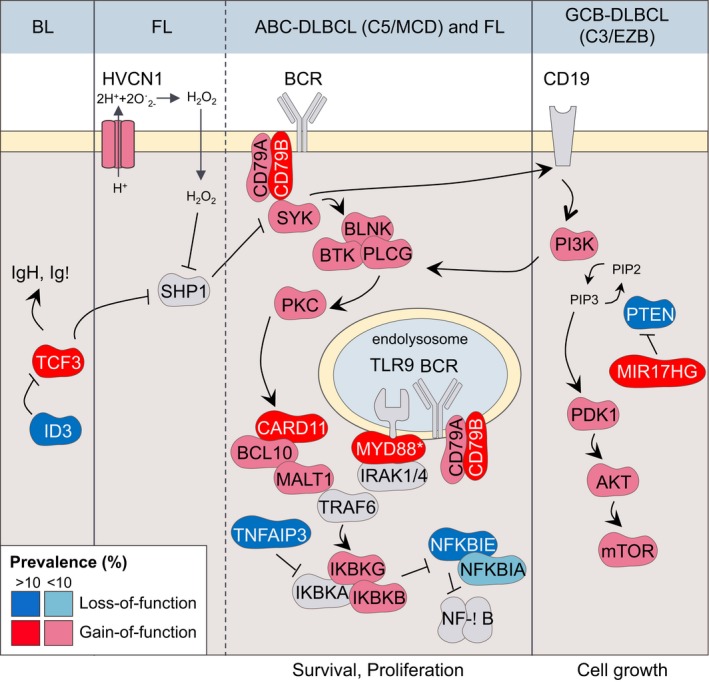
Aberrant BCR signaling in GC‐derived B‐cell lymphomas. BCR signaling is essential for mature B‐cell survival. Most GC‐derived lymphomas hijack BCR and/or PI3K signaling, including: BL, FL, the ABC‐DLBCL subtypes C5 and MCD, and the GCB‐DLBCL subtypes C3 and EZB. Depicted are the most frequent mutations that promote aberrant BCR/PI3K signaling activation in these lymphomas. Alterations that affect indirect modulators of the PI3K pathway such as PTEN and MIR17HG are more reminiscent of antigen‐independent “tonic” BCR signaling than antigen‐dependent “chronic‐active” BCR signaling. *MYD88 is mutated in ABC‐DLBCL but not in FL. Concurrent mutations of *CD79A/B* and *MYD88* are most common in C5 and MCD lymphomas and less so in other lymphomas. BL, Burkitt lymphoma; FL, follicular lymphoma; DLBCL, diffuse large B‐cell lymphoma

mTORC1 plays a crucial role in facilitating the generation of metabolic precursors through the tricarboxylic acid (TCA) cycle (anaplerosis) and in inducing cell growth (anabolism). mTORC1 activation is required by T cell‐selected GC B cells in the light zone to undergo further rounds of clonal expansion and mutagenesis in the dark zone.^47^ In the GC, mTORC1 can be activated either by nutrient signaling, which involves RagA/C‐dependent sensing of intracellular amino acids, or by T cell‐induced PI3K activation (Figure** 3). *RRAGC* (RagC) mutations are found in ~17% FL cases and co‐occur with mutations in either *ATP6V1B2* (11.3%) or *ATP6AP1* (9.9%), two components of the vacuolar ATPase proton pump (v‐ATPase).^109^, ^121^, ^122^ RagA, RagC, and v‐ATPase participate in a supercomplex at the surface of the lysosome to activate mTORC1 under nutrient sufficiency. *RRAGC* mutations cause gain‐of‐function effects that activate mTORC1 regardless of amino acid deprivation.^121^ Notably, *RRAGC* mutations frequency is 13.8% at diagnosis but 25.5% at relapse,^121^ suggesting that *RRAGC* mutations may provide a long‐term advantage to indolent FL. Deletions of *SESTRIN1*, which encodes an mTORC1 inhibitor, are mutually exclusive with *RRAGC* mutations, in support of a key role for mTORC1 activation in FL.^123^ mTORC1 may also be aberrantly activated in GCB‐DLBCL (especially C3/EZB) through activating mutations of PI3K/Akt/mTOR pathway genes, inactivating mutations of *PTEN*, mutations of *mTOR*, and amplification of *MIR17HG*, which encodes a *PTEN‐*targeting miRNA (Figures** 3 and 4).^58^, ^81^ In the normal GC context, constitutive mTORC1 activation (through deletion of the mTORC1 inhibitor Tsc1 or constitutive activation of RagA) promotes a temporary clonal expansion in the dark zone.^47^ However, this is followed by the progressive extinction of mTORC1‐constitutively active GC B cells, which feature a competitive disadvantage due, at least in part, to a failure to undergo affinity maturation.^47^ Therefore, while mTORC1 activation must be transient to support the GC reaction, constitutive mTORC1 activation seems to be highly supportive of lymphoma survival.

### 8.2 Rewiring of metabolic programs

Germinal center B‐cell lymphomas derive from cells that are highly regulated at the metabolic level. For example, BCR signaling can stimulate metabolic activity but only for a limited duration, and ex vivo BCR‐activated B cells require a second signal, such as T‐cell help, to avoid severe mitochondrial dysfunction and apoptosis.^124^ Such signal in the GC light zone possibly lies in the concomitant induction of MYC and mTORC1 that occurs upon selection by T_FH_ cells and promotes anabolism and cell survival.^14^, ^15^, ^47^ Furthermore, GC B cells transit through different micro‐compartments and must adapt by adjusting the way they utilize available energy sources. Lymphoma B cells have hijacked these metabolic rewiring capacities of GC B cells to survive.

B‐cell lymphomas can develop dependency on serine/threonine protein phosphatase 2A (PP2A) to switch glucose carbon usage from glycolysis to the PPP (Figure** 3).^125^ High expression levels of the key PPP enzyme G6PD in DLBCL patients associate with poor prognosis.^125^ Furthermore, pharmacological inhibition of PP2A or G6PD induces cell death in the DLBCL cell line OCI‐Ly10, with a strong synergistic effect of combined inhibition.^125^ PP2A inhibition or PP2A plus G6PD inhibition also increases survival of mice engrafted with primary human DLBCL cells.^125^ In normal B cells, PPP is normally kept low through the action of the transcription factors PAX5 and IKZF1 that specifically repress *G6PD* and other PPP enzymes. However, BCR activation can stimulate Glut1‐mediated glucose uptake and redirect, as cells progress through G1/S, glucose usage from primarily glycolysis to the PPP.^32^ This generates NADPH, which provides antioxidant protection to proliferating cells. Furthermore, glutamine might be used in place of glucose to replenish the TCA cycle in activated B cells, and the imported glucose would instead serve ribonucleotide biosynthesis through the PPP (Figure** 3).^30^

Diffuse large B‐cell lymphomas also develop addiction to the mitochondrial protein deacetylase SIRT3 regardless of DLBCL mutation profile or cell‐of‐origin.^126^ SIRT3 stimulates glutaminolysis by directly activating mitochondrial glutamine dehydrogenase (GDH) to enhance TCA activity and generate alpha‐ketoglutarate (aKG) (Figure** 3). *Sirt3* depletion yields reduced abundance of TCA metabolites and is rescued by GDH overexpression or addition of an aKG analog (to bypass glutaminolysis).^126^ Sirt3 knockout impairs BCL2‐driven lymphomagenesis but has no effect on normal GCs.^126^ Hence, transformed B cells develop SIRT3 non‐oncogene addiction to satisfy their metabolic needs. Because uncontrolled growth requires continuous supply of metabolic precursors, chromatin, with its abundance in acetyl and methyl groups, may represent a storage depot for such precursors in starving lymphoma B cells. The resulting depletion of chromatin modifications, however, would affect gene expression, in a similar way as somatic mutations that inactivate chromatin‐modifying enzymes (eg, *CREBBP* mutation).

### 8.3 Adaptation to low nutrient and oxygen levels

Germinal center B cells are exposed to reduced oxygen availability in the light zone and depend on the serine/threonine protein kinase Gsk3 to survive under hypoxic conditions in vivo.^31^ In the context of lymphoma, however, BCR‐dependent inhibition of GSK3 instead provides a competitive advantage under nutrient restrictive conditions, by helping to sustain the TCA cycle.^127^ BCR‐deleted tumor B cells lose their competitiveness, likely due in part to their need to break down several carbon sources (glucose, pyruvate, glutamine) to maintain TCA cycle fueling.^127^ BCR loss and *RAS*‐activating mutations are common in BL and constitutive RAS activation rescues loss of competitiveness of BCR‐deleted cells, suggesting that combining treatments to target both BCR‐proficient cells and cells that can bypass BCR deficiency in BL might be beneficial.^127^

Finally, both hypoxia and mitochondrial dysfunction can impair aKG production, leading to reduced activity of aKG‐dependent enzymes (such as TET and KDM proteins) that results in DNA and histone hypermethylation, as well HIF1‐alpha stabilization.^128^, ^129^, ^130^, ^131^ HIF1‐alpha is the master transcriptional regulator of the adaptive response to hypoxia and is constitutively stabilized in many DLBCLs and FLs.^132^ Together with MYC, HIF1‐alpha promotes aerobic glycolysis, known as the Warburg effect, instead of aerobic oxidation. This results in lactate production and is rather inefficient in producing ATP, but helps to create biomass (Figure** 3).^133^ Therefore, response to hypoxia‐induced metabolic imbalances might facilitate anabolism in GC‐derived lymphoma cells.

## 9 DISRUPTION OF SIGNALING PATHWAYS

Signal transduction is hijacked in lymphomas to promote survival of cells that would otherwise be negatively selected in the GC. As detailed in Table** 1, a large number of signal transducers are mutated or translocated in lymphomas of GC origin, including receptors, adapter proteins, kinases, phosphatases, ubiquitin ligases, GTPases or ultimately, transcription factors.^57^, ^58^, ^81^, ^106^ Although mutation frequency of most individual genes is low, when these signal transducers are grouped in pathways they can be subtype defining. Perhaps the paradigm for this would be the concurrent constitutive activation of the BCR and TLR pathways in extranodal ABC‐DLBCL (C5/MCD).^57^, ^58^, ^81^, ^106^

### 9.1 BCR and PI3K pathways

Antigen recognition triggers “active” BCR and PI3K signaling.^134^ However, B cells can also receive “tonic” BCR signaling, which is antigen‐independent and can be rescued by PI3K activation alone. This tonic BCR signaling is typical of B1 and follicular B cells (reviewed in^135^) but both active and tonic BCR signaling can be hijacked by GC‐derived lymphomas to promote survival.

1. Active BCR signaling. Many lymphomas manifest “chronic‐active” BCR signaling that is reminiscent of antigen‐dependent BCR and PI3K signaling. This phenomenon was first demonstrated in ABC‐DLBCL using a loss‐of‐function RNA interference screen to identify dependence on BCR signaling mediators.^136^ Following this effort, a number of reports confirmed the presence of somatic mutations in BCR pathway genes such as *TNFAIP3*,* CARD11*, and *CD79A/B* (Figure** 4).^137^, ^138^, ^139^ For example, FL and ABC‐DLBCL (C5 and MCD cases) preferentially carry mutations in genes characteristic of active BCR signaling (eg, *CD79A/B* or *CARD11*).
2. Tonic BCR signaling. BL and GCB‐DLBCL (C3 and EZB cases) instead present alterations of indirect modulators of the PI3K pathway (eg, *PTEN* loss or *MIR17HG* amplification) that are more related to “tonic” BCR signaling (Figure 4). In support of this notion, two‐thirds of BL cell lines show reduced survival after *CD79B* or *SYK* knockdown but remain insensitive to (further downstream) *CARD11* knockdown or IKK pharmacological inhibition (Figure** 4).^83^

On the other hand, C1 and BN2 cases feature mutations in *TNFAIP3*,* TNIP*,* BCL10* and *PRKCB* and are devoid of *CD79A/B* mutations, which is indicative of non‐canonical NF‐κB signaling.^58^, ^81^ This fits the hypothesis that C1 and BN2 cases are of marginal zone origin and suggests that deregulation of BAFF or CD40L signaling is critical to these cells, since they normally mediate marginal zone B‐cell activation and survival.^140^

### 9.2 TLR pathway

Toll‐like receptors recognize bacterial or viral components and promote NF‐κB activation, transcription of inflammatory cytokines and type I interferon (IFN) responses.^141^ Although TLR signaling is not required for GC formation, it enhances the magnitude of the GC response and of high‐affinity BCR selection. This occurs via a MYD88‐dependent mechanism that leads to preferential IgG2a/c isotype switching.^142^, ^143^ MYD88 is an adapter protein that plays a critical role in signal transduction from TLR and IL‐1 receptors. *MYD88* is frequently mutated in the C5/MCD DLBCL subtypes, with about 50% of the patients harboring the MYD88 L265P mutation.^58^, ^81^, ^144^ MYD88 forms a multiprotein supercomplex in CD79B/MYD88‐mutant cells along with TLR9 and the BCR on endolysosomes (Figure 4).^145^ This “My‐T‐BCR” supercomplex co‐localizes with mTOR, driving pro‐survival NF‐κB and mTOR signaling. Notably, presence of this supercomplex in patient samples was predictive of response to BTK inhibitors.^145^ *MYD88* mutations are also present in 15% of C1 DLBCLs but they are almost exclusively non‐L265P in this group.^58^ Although L265P and non‐L265P MYD88 mutations differentially affect binding and phosphorylation of IRAK1, they all trigger NF‐κB signaling.^144^ MYD88 non‐L265P mutations have also been reported in transformed FL^146^; however, *MYD88* mutations were not found in GCB‐DLBCL or BL.^144^

### 9.3 NOTCH pathway

NOTCH receptors are proteolytically cleaved upon activation, releasing the intracellular fragment to shuttle to the nucleus and regulate expression of specific genes. These receptors are involved in a multitude of developmental and cell differentiation processes. NOTCH1 and 2 are expressed in mature B cells while NOTCH ligands (Jagged and Delta‐like) are expressed by stromal cells. Jagged‐1, for example, is expressed by dendritic cells that nurse GC B cells and protect them from apoptosis.^147^ NOTCH2 is expressed in resting B cells and drives marginal zone differentiation, but is repressed by BCL6 in GC B cells.^86^ Expression of a constitutively active from of NOTCH2 in GC B cells suppresses GC formation, favors marginal zone differentiation, and induces growth suppression of GC‐derived lymphoma cells.^86^ C1 and BN2 lymphomas present the largest proportion (73%) of mutations affecting the NOTCH2 pathway (eg, *NOTCH2*,* SPEN*, and *DTX1*).^58^, ^81^ Most of these cases were described as “unclassified” based on gene expression profiling and are now postulated to be transformed marginal zone lymphomas and therefore of extra‐follicular (non‐GC) origin.^58^, ^81^ Consistent with this, C1 cases display low AICDA mutational signature activity.^58^ On the other hand, NOTCH1 is upregulated upon B‐cell activation and necessary for differentiation into antibody‐producing cells, in synergy with BCR signaling and CD40 or BAFF co‐stimulation.^148^, ^149^ *NOTCH1* is affected by gain‐of‐function mutations in the N1 group of DLBCLs,^81^ and in BL.^150^ FL patients exhibit low‐frequency mutations in all four NOTCH genes *NOTCH1* (4%), *NOTCH2* (4%), *NOTCH3* (5%), and *NOTCH4* (4%), as well as in the related genes *DTX1* (6%) and *SPEN* (3%). Overall, 18% of FL patients present alterations in the NOTCH pathway.^122^ However, the functional and therapeutic relevance of NOTCH in lymphomas remains uncertain.

### 9.4 JAK/STAT pathway

The JAK/STAT pathway mediates signal transduction downstream of cytokines, including IL‐4 and IL‐21, and plays essential roles in the GC reaction. IL‐4 and IL‐21 cooperate to maintain high levels of BCL6 expression in a STAT6‐ and STAT3‐dependent manner, respectively.^151^ Loss of B‐cell intrinsic IL‐4 signaling via STAT6 knockout reduces the magnitude of the GC response, while loss of IL‐21 via IL‐21R knockout reduces its duration.^151^ Concomitant loss of IL‐4 and IL‐21 signaling (via STAT6 and IL‐21R double knockout) almost completely abolishes the GC response.^151^ IL‐4 can also upregulate CD79A/B expression and increase BCR signaling through STAT6‐mediated transcription.^152^ Therefore, STAT6 is an important mediator of the GC reaction initiation and maintenance. In fact, 49% percent of EZB patients carry activating mutations or amplification of *STAT6* or deletions of its negative regulator *SOCS1*.^81^ A smaller proportion of FL patients also carry mutations in *STAT6* (8%‐12%) and *SOCS1* (12%). Constitutive activation of these genes is meant to enhance the GC response and prevent apoptosis, in favor of lymphoma survival and proliferation.

### 9.5 Gα migration pathway

Another pathway frequently mutated in GC‐derived lymphomas is the GC homing pathway involving S1PR2 and GNA13. Sphingosine‐1‐phosphate (S1P) acts via its receptor S1PR2 and the G‐protein GNA13 to inhibit migration of GC cells and control their growth.^153^ GC confinement of GC B cells is lost upon *GNA13, S1PR2*, or *P2RY8* (another S1P receptor expressed on GC B cells) loss‐of‐function, leading to dissemination of GC‐origin lymphomas.^154^ Accordingly, *GNA13* is mutated in 30% of GCB‐DLBCL^106^ and 15% of BL.^81^, ^150^ By genetic DLBCL subtypes, *SP1R2* and *GNA13* are disrupted in 38% of EZB cases,^81^ *GNA13* is frequently altered in C3, and both *GNA13* and its downstream mediators *RHOA* and *SGK1* are disrupted in C4.^58^ Mutations in this pathway may enable GC B cells, which are normally largely confined to their respective follicles, to spread to other sites resulting in systemic dissemination.

## 10 EVASION FROM IMMUNE SURVEILLANCE

**Figure 5:**
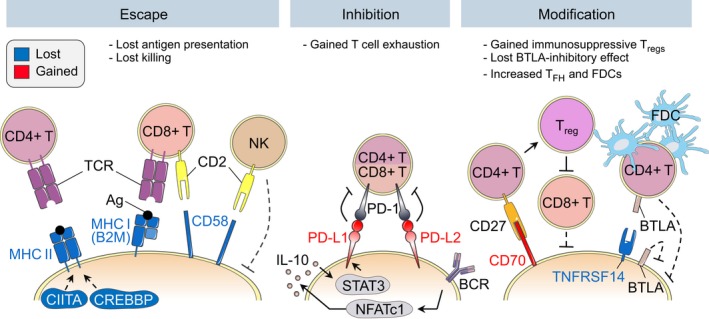
Immune surveillance evasion. Germinal center‐derived B‐cell lymphomas have evolved ways to evade antitumor immunity including escaping from immune recognition, inhibiting immune effector cells and inducing a tumor‐supportive environment. *Escape*: Loss of MHC II expression caused by *CIITA* or *CREBBP* inactivation prevents antigen presentation to CD4+ T cells. Loss of the MHC I component B2M and loss of CD58 prevent interaction with cytotoxic CD8+ T cells and natural killer (NK) cells. *Inhibition*: Some DLBCLs (C1) harbor gains of the *PD‐L1/PD‐L2* locus. Increased PD‐L1/L2 levels result in increased interaction with T cells via PD‐1, which induces T‐cell anergy. *Modification*: In FL, high CD70 levels induce differentiation of CD4+ T cells into immunosuppressive T regulatory cells that inhibit cytotoxic CD8+ cells. DLBCLs instead carry *CD70*‐inactivating mutations that might contribute to reduced interactions with antitumor T and NK cells, which also express CD27. TNFRSF14 normally interacts with BTLA (B and T lymphocyte attenuator), which regulates B‐cell expansion in the GC. Loss of *TNFRSF14* might therefore contribute to uncontrolled proliferation. It was also linked to increased recruitment of tumor‐supportive follicular T‐helper (T_FH_) cells and follicular dendritic cells (FDCs)

Upon transformation, aberrant B cells may express nonself or neo‐antigens that would allow other immune effector cells, such as T cells, natural killer (NK) cells and macrophages, to detect and eliminate them. To escape immune surveillance, lymphoma B cells have developed ways to (a) hide from the immune system, (b) actively suppress its function, or (c) modify its nature so that it becomes supportive (Figure** 5). MHC class I (MHC I) proteins, which are present on most nucleated cells, mediate presentation of self, nonself and neo‐peptides to cytotoxic CD8+ T cells. MHC class II proteins (MHC II) on the other hand, are mainly present on “professional” antigen‐presenting cells, such as B cells, and serve to activate CD4+ T cells. Selection in the GC light zone involves antigen presentation via MHC II to CD4+ T_FH_ cells and antigen recognition on FDCs through the BCR. CD40 engagement with its ligand (CD40L) on CD4+ T_FH_ cells stimulates expression of MHC II genes, adhesion molecules (such as ICAM‐1 and CD58) and PD‐L1/PD‐L2, to sustain interaction with T_FH_ and FDCs.^33^, ^34^ GC‐derived B‐cell lymphomas are thus tumors of “professional” antigen‐presenting cells and antigen presentation must be concealed for these cells to escape killing. Accordingly, MHC I/II expression is often lost in GC‐derived neoplasms.^109^, ^155^ *PD‐L1* and other important components of the immune synapse (such as CD47) are also suppressed to exacerbate the immune tolerance privileges of B cells.^156^, ^157^, ^158^

### 10.1 Escaping recognition by immune effector cells

*B2M* and *CD58* loci are often targeted by deletions and mutations in DLBCL. *B2M* encodes for beta‐2‐microglobulin, an obligatory component of MHC I surface molecules. *B2M* missense mutations affect the protein stability and cause loss of MHC I surface expression.^159^ CD58 is an immunoglobulin superfamily gene involved in the adhesion and activation of both CD8+ cytotoxic T cells and NK cells. The majority of *CD58* alterations lead to the expression of a truncated, nonfunctional protein.^159^ While CD58 expression is normally high in GC B cells compared to naive B cells, surface protein expression is lost in DLBCL cases featuring *CD58* alterations.^159^ Furthermore, CD58 expression levels modulate the cytolytic capacity of NK cells in vitro.^159^ The frequent combined loss of both *CD58* and *B2M* in DLBCL may therefore enable lymphoma B cells to escape recognition by the two arms of antitumor immunity, ie, CD8+ T cells and NK cells (Figure** 5). DLBCL and FL also overexpress CD47, a surface receptor that inhibits the phagocytic activity of macrophages and dendritic cells. A CD47‐targeting antibody used in combination with the anti‐CD20 antibody Rituximab in patients with relapsed or refractory DLBCL or FL yielded complete response in about 36% of patients.^158^, ^160^

MHC II proteins are repressed in the GC dark zone through ubiquitination‐ and epigenetic‐dependent mechanisms, but are induced in the light zone.^33^, ^161^ In DLBCL, malignant B cells become unable to express MHC II through multiple mechanisms (Figure** 5). For example, they inactivate *CIITA*, a critical activator of MHC class II genes through translocations, deletions, or inactivating somatic mutations.^58^, ^81^, ^162^, ^163^ BCL6 also mediates direct repression of MHC class II genes, *CIITA* and *CD74* (an MHC class II co‐receptor), through recruitment of SMRT/HDAC3 complexes.^88^ In addition, *CREBBP* mutation results in enhanced and sustained suppression of MHC class II levels in FL and DLBCL.^109^ Mono‐ and bi‐allelic deletions of *CREBBP* increase the proportion of GC B cells and these GC B cells feature reduced MHC II protein levels.^164^ In line with this, *CREBBP*‐mutant lymphomas associate with reduced T‐cell proliferation and T‐cell infiltration in FL.^109^ HDAC3‐specific inhibitors can rescue expression of MHC II, CIITA, and CD74 in *CREBBP*‐mutant lymphoma cells and restore the ability of tumor‐infiltrating T cells to kill DLBCL cells in an MHC class II‐dependent manner.^165^ Somatic mutation of *EZH2* is associated with profound silencing of both MHC I and MHC II genes, and EZH2‐mutant lymphomas in both mouse and humans manifest reduced expression of these genes and a reduction in lymphoma infiltrating CD4 and CD8 T cells.^114^ This repression is due at least in part to increased levels of the H3K27me3 repressive mark at antigen presentation genes, an effect that can be overcome by EZH2 inhibitors.^114^ Finally, MHC class II expression may also be affected by the differentiation status of the cells, as seen in the more plasmablastic ABC‐DLBCLs that also feature low MHC II levels.^166^

### 10.2 Inhibiting antitumor immunity

Gaining surface markers that inhibit T cells, NK cells, and macrophages is another way to evade antitumor immune surveillance. Effector T cells express the PD‐1 receptor on their surface, which upon binding to its cognate ligands PD‐L1 and PD‐L2 on the surface of antigen‐presenting cells, induces T‐cell anergy or “exhaustion.” The PD‐1‐PD‐L1/PD‐L2 axis has therefore gained great interest for anticancer therapeutic intervention.^167^ However, most FLs and DLBCLs express relatively low levels of PD‐L1, which may explain the relatively poor performance of checkpoint inhibitors in these tumors as compared, for example, to Hodgkin lymphomas.^168^ One exception to this may be the ABC‐DLBCL C1 subtype that specifically harbors gains, amplifications, and translocations of the *PD‐L1/PD‐L2* locus associated with increased expression.^58^, ^169^

PD‐L1 levels may be increased through other mechanisms as well. A recent study showed that NFATc1 activation downstream of BCR signaling was found to be responsible for IL‐10 secretion and subsequent STAT3‐dependent induction of *PD‐L1* expression (Figure** 5).^170^ NFATc1 knockdown or treatment with BTK inhibitors to inhibit BCR signaling reduced IL‐10 secretion. IL‐10 receptor is a therapeutic target in DLBCL^171^ and an IL‐10 receptor‐neutralizing antibody prevented STAT3 activation (phosphorylation) and induction of PD‐L1 protein expression.^170^ Levels of activated STAT3 also correlate with PD‐L1 protein expression in primary DLBCL.^170^ Therefore, BTK inhibition, NFAT targeting, or IL‐10 blocking could potentially be used to improve the efficacy of T cell‐directed therapies.

Finally, PD‐L1 is co‐regulated with MHC I/II through IFNγ response pathways, which are suppressed by BCL6 in GC B cells, an effect that is again reinforced by *CREBBP* mutation.^165^, ^172^ HDAC3 inhibitors rescue suppression of IFNγ response yielding upregulation of PD‐L1 and MHC I/II.^165^, ^173^ DNA methyltransferase inhibitors also induce IFNγ responses and are currently being tested in clinical trials. These approaches may restore PD‐L1 and MHC I/II at the same time thus providing a rationale for combined treatments with immune checkpoint inhibitors.

### 10.3 Inducing a tumor‐supportive environment

*TNFRSF14* encodes a receptor for the B‐ and T‐lymphocyte attenuator (BTLA) protein and is often deleted in FL patients and lost in C3/EZB DLBCL. Deletion of BTLA on T_FH_ cells results in increased IL‐21 production and GC B‐cell expansion, suggesting that interaction of TNFRSF14 on GC B cells with BTLA on T_FH_ cells inhibits uncontrolled GC development.^174^ In a mouse model of BCL2‐driven lymphoma, *TNFRSF14* knockdown accelerated disease development, with an increased proportion of T_FH_ and FDCs in the tumor microenvironment (Figure** 5).^175^ It is proposed that TNFRSF14 and BTLA could also interact in *cis* on the same lymphoma B cell.^176^ In line with this, CAR‐T cell delivery of a soluble TNFRSF14 protein to CD19+ B cells yield enhanced antitumor efficacy in a lymphoma xenograft model, as compared to CD19‐directed CAR‐T cells only.^175^ Hence, TNFRSF14‐BTLA interaction can induce cell autonomous growth inhibitory effects and although it requires BTLA expression to be maintained, engineered immune cells could be used for targeted therapy.

Lymphoma cells can also recruit and “reeducate” surrounding cells to their advantage, for example, by modulating CD70 expression levels. CD70 is the ligand for CD27, a tumor necrosis factor receptor (TNFR). In FL, high CD70 levels on lymphoma cells can increase FOXP3 levels and skew differentiation of CD4+ T cells into immunosuppressive T regulatory cells, which can negatively regulate intratumoral cytotoxic CD8+ T cells (Figure** 5).^177^, ^178^ FL retains a dependency on the GC environment and involves extensive cross‐talk between malignant and normal cells to build a tumor permissive milieu.^179^ This is not the case for DLBCL however, in which *CD70* genetic alterations are instead inactivating mutations.^58^, ^81^ The advantage of losing CD70 expression remains to be determined, but it is possible that loss of CD70‐CD27 binding in the DLBCL context can alleviate the interaction of tumor B cells with potential antitumor T or NK effector cells. Certain GC‐derived lymphomas evolved further mechanisms to rewire the environment to their advantage. These include (a) recruiting macrophages or regulatory T cells with immunosuppressive functions,^180^, ^181^ (b) impairing the motility of infiltrating CD8+ cytotoxic and CD4+ T cells by the modulation of their gene expression program in FL,^182^ and (c) transforming tumor‐infiltrating T_FH_ cells into follicular regulatory T (T_FR_) cells by changing their gene expression profiles so that they become highly supportive of malignant B cells.^183^

## 11 SHIFTING THE THERAPEUTIC PARADIGM FOR GC‐DERIVED LYMPHOMAS

Current chemoimmunotherapy regimens for GC‐derived lymphomas provide relatively favorable response rates in newly diagnosed DLBCL, FL, and BL. Although patients with refractory or relapsing disease still have a poor prognosis, novel therapeutic options such as CAR‐T cells may rescue a subset of these cases. Patients with indolent lymphomas are often observed and therapy withheld, until there is evidence of disease progression. All of these treatments are empiric, and not targeted to disease driver mechanisms. However, given current advances in scientific understanding of these diseases and availability of targeted therapies, it is now possible to envision a future where precision therapy relevant to the underlying genetics and tumor mechanisms could replace the current standard treatments and yield superior outcomes. It is appealing to consider such treatments as early intervention for patients with indolent disease like FL that nonetheless has the risk of transforming to a refractory aggressive disease. Some of these new modalities are described below.

### 11.1 Epigenetic therapy

The realization that most GC‐derived lymphomas manifest mutations of epigenetic modifiers provides a firm rationale for precision epigenetic therapy. Until recently, the only epigenetic targeted therapy available has been DNA methyltransferase inhibitors. These drugs are appealing for DLBCL since (a) they require incorporation into DNA and as DLBCLs are rapidly proliferative, uptake of drug occurs rapidly; (b) hypermethylation and silencing of genes such as *SMAD1* mediate DLBCL chemotherapy resistance and this can be demonstrably reversed in human DLBCL patients using DNA methyltransferase inhibitors; and (c) at low demethylating doses, the compounds are well tolerated by humans in combination with chemotherapy.^184^ Additional potential benefits include the induction of IFN signaling pathways to enhance immunogenicity,^173^ although this has not yet been proved in the DLBCL context. Phase I/II trials of DNA methyltransferase inhibitors have yielded encouraging results.^185^

Pan‐HDAC inhibitors are often described as “epigenetic therapy” in the literature but it is the view of our group that there is no clear evidence that their antitumor effects are related to effects on the epigenome. Thousands of proteins throughout the cell are regulated by lysine acetylation and the effects of these drugs are too pleiotropic to interpret or recommend as an approach to target gene repression. Indeed, although HDACs are repressors, the gene expression signatures induced by HDAC inhibitors are not skewed toward transcriptional activation. The drugs are fairly toxic and cannot be pushed to full target engagement in humans, and toxic drugs are more difficult to use in combination therapy settings. Primary T‐cell lymphomas as well as normal T cells are sensitive to these drugs, which also raises concerns about their use in combination with immunotherapy regimens.^165^, ^186^

Perhaps a more precise approach for targeting the epigenome is provided by specific EZH2 inhibitors, which are particularly effective in suppressing EZH2 mutant lymphoma cells and EZH2 mutant lymphomas in humans.^16^, ^187^ Wildtype DLBCLs and FLs may also respond, given that EZH2 is an essential protein in GC B cells from which lymphomas arise. Important considerations with these agents include that (a) sustained target suppression is probably important, and may be difficult to achieve especially in more aggressive tumors, (b) aside from *EZH2* mutation, there is no biomarker to predict which EZH2 wildtype patients might respond, and (c) these drugs are mostly cytostatic, so that combination with other agents is needed to achieve maximal effect. Among these, combination with BH3 mimetics was shown to be highly synergistic and is well suited to the setting of EZH2 mutant FLs and EZB DLBCLs.^16^ Given the role of mutant EZH2 in suppressing MHC I/II, it seems likely that these drugs would greatly enhance the efficacy of immunotherapies such as checkpoint inhibitors.^114^ Although current regimens involve continuous and prolonged dosing, this can lead to the development of resistance and may not be necessary if the drug is used in more brief cycles in combination.^188^ It will be necessary to follow the outcomes of clinical trials with different EZH2 inhibitors of varying potency, mechanism of action and pharmacokinetics to fully understand the optimal manner in which to use this modality in the clinic.

HDAC3 selective inhibitors are of interest for precision therapy of patients with CREBBP‐mutant lymphomas. HDAC3 is mostly contained in SMRT and NCOR corepressor complexes, which in GC B cells are almost uniquely associated with BCL6.^87^ HDAC3 selective inhibitors yield transcriptional signatures in DLBCL cells consisting almost entirely of gene activation, consistent with their effects being truly epigenetic.^165^ The major benefits of these compounds is expected to be in restoring anti‐lymphoma immunity through induction of MHC I/II, with potential synergy in combination with checkpoint inhibitors, and in their ability to achieve full target suppression without invoking the toxicity associated with pan‐HDAC inhibitors.^165^

KMT2D‐mutant lymphomas appear to be addicted to the KDM5 family of histone demethylases^111^ (and Jude Fitzgibbon, personal communication). KMT2D‐mutant lymphoma cells become hyper‐responsive to CD40 agonists when treated with KDM5 inhibitors, yielding potent synergy and enhanced efficacy in vivo.^111^ As several CD40 agonists are also in clinical trials for DLBCL, this is an appealing rational therapy for KMT2D mutant FLs and DLBCLs. BET domain protein inhibitors downregulate genes with expression driven by BRD4‐dependent gene enhancers, and have activity against DLBCL cell lines in vitro and in vivo.^189^ They cannot really be viewed as precision therapy for DLBCL since there currently is no biomarker or genetic lesion that predicts for selective response to these agents, and their actions are fairly pleiotropic. Further study is needed to understand their role and utility in GC‐derived lymphomas. Finally, drugs targeting the symmetric arginine methyltransferase PRMT5 are also highly active against DLBCL cells, which may be partly related to PRMT5 acting as a corepressor for BCL6.^190^, ^191^ As PRMT5 is also involved in RNA‐splicing, the actions of these inhibitors are likely also pleiotropic.

### 11.2 Targeting metabolic vulnerabilities

Lymphomas must adapt to high energy demands to sustain survival and proliferation under scarce nutrient and oxygen conditions.^192^ These adaptations represent a potential route for targeting lymphoma B cells while sparing normal GC B cells. Recent studies have identified such vulnerabilities. One such example is dependency of DLBCL on SIRT3 that can be suppressed using mitochondrial‐targeted class I sirtuin inhibitors.^126^ BL develops dependency on BCR‐dependent GSK3 inhibition and evolve mechanisms to bypass BCR loss such as *RAS* mutations.^127^ Combination therapies that target both BCR‐positive and ‐negative tumor cells may therefore show better efficiency. In DLBCL and other B‐cell malignancies, targeting the rewiring of glucose usage from glycolysis to the PPP also represents a possible therapeutic option.^125^ In addition, certain DLBCLs defined by gene expression profiling manifest addiction to glycolysis, while other “OxPhos‐DLBCLs” depend on the electron transport chain (ETC) activity.^193^, ^194^ OxPhos‐DLBCLs were reported to require mitochondrial palmitate oxidation to produce ATP,^193^ or the mitochondrial translation machinery to raise ETC protein levels.^194^ Inhibition of the mitochondrial fatty acid oxidation^193^ or mitochondrial translation machinery^194^ reduced the viability and proliferation of OxPhos‐DLBCL cells. Overall, these intriguing results point to the need for gaining deeper knowledge of the regulation of nutrient sensing and metabolism in normal and malignant GC B cells in order to develop precise therapeutic strategies against metabolically dependent GC‐derived B‐cell lymphomas. Finally, it is also important to note that tumor cells and antitumor T cells evolve in the same nutrient‐deprived microenvironment and compete for energy sources. We speculate that lymphoma B cells that have undergone metabolic rewiring are better fit than normal immune T cells in these conditions, and when combined with genetic lesions that favor antitumor immune escape (such as *TNFRSF14* loss, *CD70* gain, etc.) might lead to a situation highly advantageous for immune escape, but vulnerable to combination of metabolic and immunotherapies.

### 11.3 Therapeutic targeting of aberrant signal transduction

The PI3K pathway is implicated not only in lymphomas but almost every other cancer, leading to the development of many PI3K inhibitors with distinct isoform selectivity. Among these, Idelalisib, Copanlisib and Duvelisib demonstrated efficacy and are FDA‐approved for the treatment of FL patients, while mTOR inhibitors have shown 38%‐54% overall response rate (ORR) in phase II clinical trials in relapsed FL patients (reviewed in^195^). Despite the pathway being altered, response rates in aggressive DLBCL as well as transformed FL were moderate for PI3K inhibitors.^196^, ^197^ This could be due, at least in part, to the largely cytostatic effect of these drugs and to the cases mutational status not accounted for. Whether specific subtypes of DLBCL could particularly benefit from this treatment is currently unknown. In vitro studies suggest that PI3K inhibition has greater potency in ABC‐DLBCL, while AKT inhibition is most effective in PTEN‐deficient (mostly GCB‐) DLBCL.^198^ mTOR inhibition, on the other hand, showed moderate efficacy in DLBCL with 28%‐29% or 37.5% ORR, alone or in combination with the anti‐CD20 monoclonal antibody Rituximab, respectively.^195^ Based on their genomic profile, BL patients would also be expected to respond to mTOR inhibition.

The BTK inhibitors Ibrutinib and Acalabrutinib have shown promising results in relapsed or refractory FL, with a 37.5% ORR in a phase II clinical trial.^199^ Notably, none of the five patients who carried *CARD11* mutations responded and the highest response rates were observed among Rituximab‐sensitive patients.^199^ A phase 1b study combining BTK inhibitor and Rituximab has shown remarkable ORR in treatment‐naive FL patients (92% ORR, with 31% CR) but only a moderate response in relapsed or refractory patients (39% ORR).^200^ BTK inhibition was also moderately potent in ABC‐DLBCL (37% ORR) with the highest response in patients carrying concomitant BCR‐activating and *MYD88* mutations (80% ORR),^201^ which are canonical features of extranodal ABC‐DLBCL. Adding BTK inhibitor to the standard DLBCL chemoimmunotherapy R‐CHOP regimen in non‐GCB‐DLBCL did not improve survival^202^ but other combinations might prove to be more beneficial. BTK inhibition has proven quite successful when used as a single agent in PCNSL (primary central nervous system DLBCL) and secondary CNSL,^203^, ^204^, ^205^ with clinical response seen in 78% of CNS lymphoma patients in a phase II study.^204^ An Ibrutinib/Methotrexate/Rituximab combination has shown manageable toxicity and promising results in relapsed/refractory CNS lymphomas with 80% clinical response rate in a phase Ib trial.^204^

Targeting further downstream components of the BCR pathway is desirable in order to bypass BTK inhibitor resistance conferred by mutations of genes such as *PLC*γ, *CARD11*, and *BCL10*. Along these lines compounds that target the MALT1 paracaspase are highly effecting in suppressing ABC‐DLBCLs in vitro and in vivo.^206^, ^207^, ^208^, ^209^ MALT1 is a key mediator of the BCR signaling to NF‐κB and is essential in the development of ABC‐DLBCL and other lymphomas including mantle cell lymphoma, primary effusion lymphoma, and chronic lymphocytic leukemia (CLL).^136^, ^210^, ^211^, ^212^, ^213^, ^214^ Moreover, MALT1 inhibition was shown to be effective in CLL patients, even those with mutations of *BTK*,* PLCy*, and *CARD11* that cause Ibrutinib resistance.^214^

Regarding TLR signaling, IRAK4 inhibitors show activity in preclinical studies in ABC‐DLBCL cell lines^144^ and PDXs.^215^ A phase 1b clinical trial of one such compound is currently underway.^215^ JAK2 inhibitors, which are commonly used to treat myelofibrosis, are being tested in clinical trials for diverse lymphoma subtypes. However, a recent study reported a 20‐fold increase (0.3%‐5.8%) in the prevalence of aggressive B‐cell lymphomas in myelofibrosis patients treated with vs without JAK2 inhibitors. In the three patients surveyed, the B‐cell clone was a preexisting clone that grew while on treatment.^216^ This demands further studies and stresses the need for close monitoring of patients under JAK2 inhibition treatment for secondary malignancies.

It is important to take into consideration that signal transduction exhibits enormous plasticity. Targeting of one pathway is usually not curative but rather tends to select for cells with different dependencies or to induce feedback compensatory mechanisms that maintain cell survival. We would therefore advocate for the concomitant targeting of more than one signaling mediator (eg, blocking a pathway and its feedback mechanisms) or cell functions (eg, proliferation blockage and apoptosis induction; proliferation blockage and immune checkpoint unleash). For example, it was shown that tumor‐specific Hsp90 inhibitors disrupt BCR signaling at various steps, thus creating a situation where combination with BTK inhibitors yields synergistic killing of ABC‐DLBCL cells.^217^

### 11.4 Immunotherapy

The majority of immunotherapies require potent tumor antigen presentation to T cells. However, unlike normal light zone GC B cells, which are the cell‐of‐origin of FL and DLBCL, lymphoma B cells exhibit low levels of antigen presentation proteins as well as other immune defects. Low MHC class II expression has been associated with poor outcome in DLBCL, likely due to impaired immune surveillance.^155^ Therefore, therapeutic intervention to restore the immune system's antitumor activity would likely prove to be powerful in GC‐derived B‐cell lymphomas. Clinical trials for PD‐1/PD‐L1 blockade have shown some response in refractory and relapsed FL (40% ORR) and DLBCL (36%) but not as striking as in non‐GC‐derived classical Hodgkin lymphoma (87%).^168^, ^218^ A combinational approach with synergistic drugs to jointly target several immune evasion mechanisms would therefore likely be highly beneficial. More recently, clinical trials with chimeric antigen receptor (CAR)‐T cell therapy targeting CD19 cells have shown especially high response rates and durability of remission in refractory and relapsed FL and DLBCL.^219^, ^220^ Together, the advances in CAR‐T cell biology, in GC‐derived B‐cell lymphomas identity, and pathogenesis mechanisms will allow developing efficient and precise medicine for each patient, taking into account the patient's tumor unique molecular profile and using the patient's own T cells.

## 12 CONCLUDING REMARKS

Recent years have seen dramatic acceleration of our understanding of the humoral immune response and lymphomagenesis. Sequencing the lymphoma genome has illuminated many new critical regulators of the GC reaction and mechanistic studies of these mutations shed light both on normal and malignant GC B‐cell biology. Along with these advances we are nearing the point where lymphoma genomes will dictate selection of precision therapies geared to reverse the effect of specific mutations or target biologically defined patient subsets. One area of special interest will be using knowledge of how immune regulatory networks are suppressed in GC‐derived lymphomas to develop molecular targeted plus immunotherapy regimens, with real potential to eradicate disease even in the most difficult to treat lymphoma patients.

## CONFLICT OF INTEREST

AM receives research support and has consulted for Janssen Pharmaceutical. He is also a scientific advisor to KDAC pharmaceuticals.
